# Case report of restless anal syndrome as restless legs syndrome variant after COVID-19

**DOI:** 10.1186/s12879-021-06683-7

**Published:** 2021-09-23

**Authors:** Itaru Nakamura, Takao Itoi, Takeshi Inoue

**Affiliations:** 1 Tokyo Medical University Hospital, Tokyo, Japan; 2 Department of Infection Prevention and Control, Tokyo Medical University Hospital, 6-7-1 Nishishinjuku, Shinjuku-ku, Tokyo, 160-0023 Japan

**Keywords:** COVID-19, Anal, Restless, Complication, Neuropsychiatric

## Abstract

**Background:**

Coronavirus disease 2019 (COVID-19) has a broad spectrum from respiratory and nasopharyngeal symptoms, cerebrovascular diseases, impaired consciousness, and skeletal muscle injury. Emerging evidence has indicated the neural spread of this novel coronavirus. Restless legs syndrome (RLS) is a common neurological, sensorimotor disorder, but highly under diagnosis disorder. Restless anal syndrome as restless legs syndrome variant associated with COVID-19 has been previously not published. We report a case presenting with restless anal syndrome following COVID-19.

**Case presentation:**

Although a 77-year-old male with COVID-19 improved to normal respiratory function 21 days after admission and treatment of favipiravir 200 mg per day for 14 days and dexamethasone 6.6 mg per day for 5 days, the insomnia and anxiety symptoms remained. Several weeks after discharge, he gradually began to experience restless, deep anal discomfort, approximately 10 cm from the perineal region. The following features were observed in the anal region; urge to move is essential, with worsening with rest, improvement with exercise, and worsening at evening. Colonoscopy revealed internal haemorrhoids without other rectal lesions. Neurological findings including deep tendon reflex, perineum loss of sensory and spinal cord injury, revealed no abnormalities. Diabetes militias, kidney dysfunction and iron deficiency status were not confirmed. Family history of RLS and periodic limb movements were not observed. Clonazepam at 1.5 mg per day resulted in the alleviation restless anal discomfort.

**Conclusions:**

We reported a case presenting with restless anal syndrome following affection of COVID-19 as restless legs syndrome variant. This case fulfilled 4 essential features of RLS, urge to move, worsening with rest, improvement with exercise, and worsening at evening. To date, no case of restless anal syndrome associated with COVID-19 has been previously published. This case report may reflect the associative impacts of COVID-19 on the neuropsychiatric state. The long-term outcomes of neuropsychiatric conditions should continue to be monitored.

## Background

Coronavirus disease 2019 (COVID-19) is an emerging infectious disease caused by severe acute respiratory syndrome coronavirus 2 (SARS-CoV-2), which has rapidly spread worldwide. A broad spectrum of COVID-19 symptoms has been reported, including fever, cough, sore throat, dyspnoea, and the loss of smell or taste [1, 2]. Addition to respiratory and nasopharyngeal symptoms, cerebrovascular diseases, impaired consciousness, and skeletal muscle injury have been recognized in association with this disease [3]. Surprisingly, although the mechanism of COVID-19 related neuro and psychical symptoms are not fully understood, emerging evidence has indicated the neural spread of the novel coronavirus [4]. Additionally, the other multi factors, such as consequences of brain hypoxia, systemic inflammation, secondary effect of medication as well as direct invasion to nerve systems, are suspected [5].　Neuropsychial symptoms, being concurrent with and following COVID-19, has been diversely reported from delirium, confusional status, dysfunction of olfaction, dysfunction of taste sensation, acute psychosis, encephalitis, cerebral vascular event, Guillain-Barre Syndrome [4]. Thus, the spectrum of symptoms associated with COVID-19 has widened from the initially recognized respiratory symptoms to include a larger variety of symptoms than expected.

Restless legs syndrome (RLS) is a common neurological, sensorimotor disorder arising from dysfunction of the central nervous system, but highly undiagnosis disorder. The characteristic symptoms are urge to move is essential, with worsening with rest, improvement with exercise, and worsening at evening [6]. The prevalence are reported in a few% of adults in Japanese population, similar to European and American population [6–8]. RLS cases have also a various symptom form mainly legs symptom to head, oral cavity, abdomen, perineum, which are recognized as RLS variant cases [9–11]. The pathology of RLS is suggested to be from dopaminergic dysfunction on the level of dopamine receptors, and iron deficiency, kidney dysfunction, diabetes mellitus are the exacerbation factor [6].

Although the previous reports of COVID related RLS cases are limited, here, we report a case presenting with restless anal syndrome as restless legs syndrome variant following diagnosis with COVID-19.

## Case presentation

A 77-year-old male presented with sore throat, cough, and low-grade fever on day 1. The following day, a positive nasopharyngeal PCR sample was obtained. Following admission to Tokyo Medical University Hospital, mild pneumonia was detected, which was treated with ciclesonide inhalation and favipiravir 200 mg per days for 14 days following dexamethasone 6.6 mg per day for 5 days, and insomnia and anxiety were treated with zolpidem, brotizolam, and quetiapine. He did not require oxygen therapy during the in-hospital clinical course, despite a prolonged fever for 10 days, and the patient was categorized as a mild COVID-19 case. Although he improved to normal respiratory function 21 days after admission and the treatment, the insomnia and anxiety symptoms remained. Before affecting COVID-19, he had never experienced anal restless and discomfort. However, several weeks after discharge, he gradually began to experience restless, deep anal discomfort, approximately 10 cm from the perineal region. This restless anal discomfort did not improve following defecation. Exercise such as walking or running and enthusiastically playing the television game made the symptoms relief, while taking a rest made the symptom worsen. Additionally, the symptom tended to be worsen in evening. The sleep was somehow kept by taking sleeping drugs. Colonoscopy revealed internal haemorrhoids without other rectal lesions. No other bladder or rectal disturbance or erectile dysfunction was confirmed. Neurological findings including deep tendon reflex, perineum loss of sensory and spinal cord injury, revealed no abnormalities. Serum iron level was not measured, while ferritin and haemoglobin revealed 688 ng/mL (normal range; 9.0–275 ng/mL) and 15.3 g/dL, respectively. Serum creatinine and Hb A1c were 0.93 mg/dL and 5.2%. Because he suffered in the anal region; urge to move, worsening with rest, improvement with exercise, and worsening at evening without legs symptoms, we diagnosed the patient as restless anal syndrome as restless legs syndrome variant after COVID-19. The diagnosis was made by face-to-face interview and physical examination by internal medicine physician and psychiatrist familiar to RLS. Clonazepam treatment at 1.5 mg per day, which is a treatment option of restless leg syndrome recommended in guideline of Japanese society of neurological therapeutics, resulted in the alleviation of restless anal discomfort without administration of dopamine agonist. On 10 months after the treatment introduction, the symptom continues to alleviate. Family history of RLS and periodic limb movements were not observed.

## Discussion and conclusion

We reported a case presenting with restless anal syndrome following affection of COVID-19 as restless legs syndrome variant. This case fulfilled 4 essential features of RLS, urge to move, worsening with rest, improvement with exercise, and worsening at evening. Because he had never experienced anal restless and discomfort before affecting COVID-19 and the anal restless symptom developed after COVID-19, we considered that these anal restless symptoms were suggested the COVID-19 related syndrome.

This virus may spread to the central nervous system through several potential routes, including hematogenous dissemination and the destruction of the olfactory bulb [4]. Reported neuropsychiatric manifestations of COVID-19 have included delirium, confessional states, dysfunctional olfaction and taste sensation, acute psychosis, encephalitis, and acute cerebrovascular events during COVID-19 [4]. Compared with these manifestations, peripheral neuropathies and Guillain-Barre Syndrome associated with COVID-19 have been rarely reported　[12]. However, the understanding of the neuropsychiatric sequelae associated with COVID-19 remains in the initial stages, and the potential mechanisms underlying these varied symptoms are not yet fully understood. Insomnia, depressed mood, post-traumatic stress disorder, and cognitive impairment have been reported in patients after discharge from the hospital [5]. Previous reports have suggested that depressive symptoms are associated with systemic immune suppression, based on increased white blood cells and inflammatory factors [13]. The onset of neuropsychiatric symptom are reported to occur in most hospitalization patients during the disease, while time lines of some disorders and cases including Guillain-Barre Syndrome has been broad with para-infections and post-infection after a few weeks from COVID onset [12, 14].

RLS arises from dysfunction of the central nervous system leading to both sensory and motor symptoms [6]. According to the ethology study of RLS at pre COVID era, the prevalence ranges from1.0 to 4.0% in Japanese population [7, 8, 15, 16]. To the data, limited cases of COVID-19 related RLS were sporadically reported. Previous study [17, 18] reported a 36-year-old female case and a 48-year old female case of RLS during COVID-19. Although COVID related RLS itself remains rare, no case of restless anal syndrome associated with COVID-19 has been previously published.

This case fulfilled with the essential features of urge to move is essential, with worsening with rest, improvement with exercise, and worsening at evening among the specific criteria of RLS. These 4 features were essential diagnostic criteria for RLS [6] Additionally, another medical condition possibly caused by such as diabetes mellitus, iron deficiency anemia, renal dysfunction, spinal cord dysfunction, was not observed. Anorectal disorders such as anorectal tumor, fistula, or inflammation are conditions that require treatment and care, resulting in anxiety, depression or sleep problems [19]. In this case, these structural and mechanical anorectal disorder was not also observed. Although functional anorectal pain is basically observed in the cases of levator ani syndrome or proctalgia fugax, the following 4 features was able to exclude functional anorectal pain.

Because the symptom localized in legs was not observed, this case was diagnosed as a variant of RLS. Reports of RLS variant has been increased and expand the clinical spectrum. Various other parts of the body than legs can be involved from arms, abdomens, face, head, oral cavity, bladder and genital area [9, 11, 20–22]. Supportively, this case did not report daytime sleepiness, which was also supporting feature of RLS [6]. This case also had the tendency of the anxiety and impatient, but no history of panic attack. As SARS-Cov-2 can also cause digestive system disease such as diarrhea, nausea, or vomiting, medical imaging might manifest as thickening of the intestinal wall [23]. In this case, the symptom was not localized in intestine, in addition to urge to move and relief by move, which characteristic was not compatible to intestinal symptoms. Also, medical imaging showed no abnormal finding in intestine.

Clonazepam treatment at 1.5 mg per day was selected in this case, which is a treatment option of restless legs syndrome recommended in guideline of Japanese society of neurological therapeutics. International restless legs syndrome study group mention that dopaminergic treatment generally shows clinical benefit for most RLS, and failure to respond to dopaminergic treatment raise some concern about diagnostic accuracy. However, they also states that the lack of dopaminergic treatment response does not preclude as the diagnosis [6].

This case report may reflect the associative impacts of COVID-19 on the neuropsychiatric state. On the other hand, the causative relation remains unclear because of a case report. Other study cautioned mimic RLS cases even if the symptoms fulfilled the diagnostic criteria [24, 25]. Because neuropsychiatric sequelae require longitudinal observation, the long-term outcomes of neuropsychiatric conditions should continue to be monitored. COVID-19 related RLS or RLS variant may be underdiagnosed and we should pay attention to similar cases in order to clarify of relation between COVID-19 and RLS.

## Data Availability

The dataset supporting the conclusions of this article is included in the article.

## Abbreviations

COVID-19: Coronavirus disease 2019
SARS-CoV-2: Severe acute respiratory syndrome coronavirus 2
RLS: Restless legs syndrome
